# Ablation index-guided catheter ablation of incessant ventricular tachycardia originating from the anterolateral papillary muscle

**DOI:** 10.1007/s00392-021-01923-x

**Published:** 2021-11-01

**Authors:** Vanessa Sciacca, Julia Vogler, Charlotte Eitel, Karl-Heinz Kuck, Roland Richard Tilz, Christian-H. Heeger

**Affiliations:** Medizinische Klinik II (Kardiologie, Angiologie, Intensivmedizin), Universitäres Herzzentrum Lübeck, Universitätsklinikum Schleswig-Holstein (UKSH), Ratzeburger Allee 160, 23538 Lübeck, Germany

Sirs: ventricular tachycardia (VT) comprises a heterogenous group of cardiac arrhythmias. VT arising from the papillary muscles of the left ventricle was first described as a clinical syndrome in 2008 [1]. As a clinical entity, papillary muscle VT is uncommon and occurs relatively rare as a sustained arrhythmia. VT from papillary muscles mostly occur due to focal mechanisms including enhanced automaticity and triggered activity [2]. Today, catheter ablation has become a well-established therapy for symptomatic patients. In the following, we report about successful catheter ablation of incessant VT of the anterolateral papillary muscle guided by 3D mapping and ablation index only.

A 50-year-old male patient was admitted to the emergency department because of palpitations. The patient was on no regular medication and had no pre-existing conditions. A family history of cardiac diseases was not present. An echocardiography showed a normal biventricular function without wall motion abnormalities. The baseline ECG showed a tachycardia around 120–140/bpm with broad QRS complexes and regular RR intervals with right bundle branch block morphology and right axis deviation (Fig. 1A). Adenosine was administered for differential diagnosis. After 18 mg of Adenosine, the tachycardia was not terminated, turned irregular and capture beats occurred confirming the diagnosis of VT (Fig. 1B). VT morphology was compatible with an origin close to the anterolateral left ventricular (LV) papillary muscle.

**Fig. 1:**
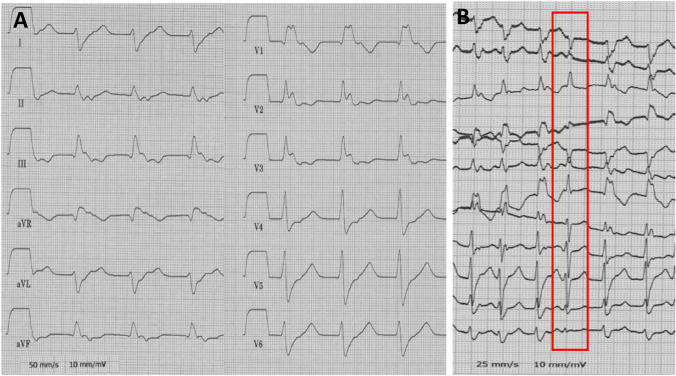
Baseline surface ECG at timepoint of admission. **A** The baseline surface ECG showed an incessant regular broad complex tachycardia with right bundle branch block morphology and right axis deviation. **B** After administration of 18 mg Adenosine, the tachycardia did not terminate but converted into an irregular rhythm. The red box indicates a capture beat during incessant ventricular tachycardia

Catheter ablation was scheduled and performed in analgosedation with propofol, midazolam and fentanyl. A decapolar catheter was advanced into the coronary sinus (CS). A DECANAV (Biosense Webster, Inc., Diamond Bar, CA, USA) mapping catheter was utilized for high-resolution multielectrode three-dimensional electroanatomical reconstruction (CARTO® 3 System, Biosense Webster) via fast anatomical mapping (FAM) and the CONFIDENSE module (Biosense Webster) of the right (RV) and LV. Mapping of the LV was conducted via a combined approach by antegrade transseptal access and retrograde transaortal access. For ablation an 8F, 3.5 mm irrigated-tip catheter ThermoCool© SmartTouch© Surround Flow catheter (Biosense Webster) was utilized. Contact force visualization (10–40 g) and ablation index (target: 600) were used to guide the ablation. Ablation lesions were visualized with the Visitag Surpoint module (Biosense Webster). Additional imaging techniques such as intracardiac three-dimensional echocardiography were not used.

At the beginning of the procedure, the clinical VT with a right bundle branch morphology, right axis deviation and irregular RR intervals was incessant. The earliest activation site was found in the anterolateral part of the LV at the area of the anterolateral papillary muscle (Figs. 2, 3, video 1). Within this area, the distal mapping signal preceded the QRS onset by 30 ms. Extended radiofrequency current ablation was performed in power-controlled mode with a limitation to 40 W. For the antegrade approach, no stable contact force was achieved on the target area. Therefore, a retrograde approach was used. A contact force of 25–40 g was achieved in this area via the retrograde approach and the VT was terminated after 7 s of radiofrequency current application via the transaortic approach. After a waiting period of 30 min, no VT or premature ventricular contractions (PVC) occurred spontaneously or were inducible by programmed stimulation. The procedure duration from groin puncture to sheath removal was 150 min. No procedural-related complications occurred. The patient was followed up for 3 months with clinical visits at the outpatient department and repetitive Holter ECG. No recurrence of VT or PVC was observed.

**Fig. 2:**
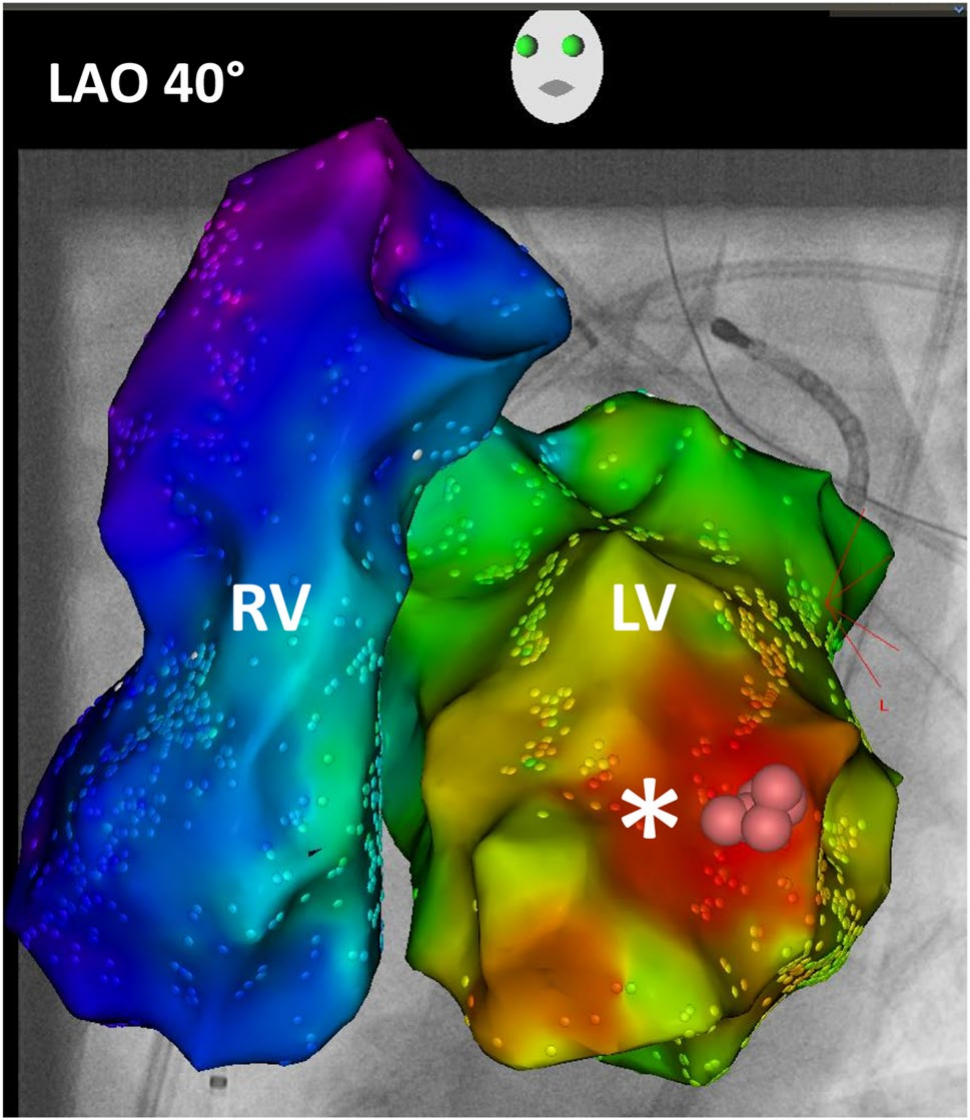
High-resolution three-dimensional electroanatomical reconstruction of the right and left ventricle. Assessment of local activation time via high-resolution multielectrode 3D electroanatomical reconstruction of the right and left ventricles with the CARTO-3 system. Earliest local activation time was found to be at the anterolateral left ventricle. The activation pattern showed a focal VT origin

**Fig. 3:**
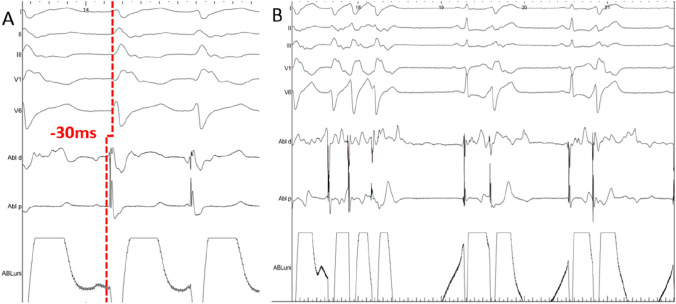
Intracardiac eletrograms. **A** Surface and intracardiac electrograms during mapping on the target area at the anterolateral papillary muscle. The distal map signal is preceding the QRS onset by 30 ms. **B** Surface and intracardiac electrograms during ablation at the anterolateral papillary muscle and termination of the VT after 7 s

VT and PVC may arise from endocavitary left or right ventricular structures. The papillary muscles of the LV are a relatively rare but well-described origin of PVC, non-sustained or sustained VT [2]. Today, radiofrequency ablation has emerged an effective therapy of VT. Nevertheless, ablation of VT from the papillary muscles is known to be challenging as the special anatomical features may lead to reduced catheter stability and hence to reduced ablation success with relatively high recurrence rates up to 71% regarding VT or PVC arising from the anterolateral papillary muscle [3, 4]. Ablation can be challenging not only due to the special anatomy of the papillary muscles but also because of a deep origin inside the PM and multiple exit sites. The implementation of 3D-mapping systems is, therefore, recommended for endocavitary ablation of PVC and VT [5]. The use of intracardiac echocardiography (ICE) may facilitate ablation of endocavitary structures such as the papillary muscles. Nevertheless, reimbursement issues and local availability of ICE may vary significantly and limit its broad usage. Cryoenergy-based ablation has been described as an adjunctive or alternative therapy when radiofrequency-based ablation for papillary muscle arrhythmia is not available or has failed [6, 7]. A definite conclusion on superiority of cryoablation-based approaches compared to the standard use of radiofrequency ablation cannot be made though due to the presence of only smaller case series. Bipolar radiofrequency-based ablation of papillary muscle PVC has recently been described as another alternative approach [8]. As the papillary muscles happen to be delicate anatomical structures within the LV concerns regarding safety of bipolar ablation approaches remain. At the present state of research, unipolar ablation represents the method of choice in the majority of cases as the use of bipolar ablation has not been evaluated in larger cohorts or prospective studies so far [9].

In the present case, we performed successful radiofrequency-based ablation of incessant anterolateral papillary muscle VT without ICE. Catheter stability and tissue contact was guided only by use of contact force and the ablation index module of the CARTO-3 system. Under guidance of 3D mapping and implementation of contact force and ablation index, a stable catheter position could be achieved and ablation was successful. Ablation index is an objective marker of lesion quality incorporating contact force, time, power and catheter stability in a weighted formula. When performing radiofrequency-based ablation of papillary muscle VT, the implementation of contact force and ablation index may be the significant factor of successful ablation due to improvement of catheter stability and vector orientation.

Ablation of PVC, non-sustained or sustained VT arising from the anterolateral papillary muscle is challenging due to the special anatomy. We demonstrate a case of successful radiofrequency-based ablation of an incessant VT arising from the anterolateral papillary muscle in an ablation index-guided procedure with antegrade and retrograde access without additional use of ICE, bipolar ablation or cryothermal energy ablation.

## Supplementary Information

Below is the link to the electronic supplementary material.

Supplementary file1 (MP4 7817 KB)

## Abbreviations

ICE: Intracardiac echocardiography
CS: Coronary sinus
LV: Left ventricle
PVC: Premature ventricular contraction
RV: Right ventricle
VT: Ventricular tachycardia
